# Crystal structure of (4-amino-3,5-di­chloro­pyridine-κ*N*^1^)dioxidobis(pentane-2,4-dionato-κ^2^*O*,*O*′)uranium(VI) methanol hemisolvate

**DOI:** 10.1107/S2056989026009618

**Published:** 2026-09-25

**Authors:** Shogo Miyazaki, Kosuke Kitase, Takeshi Kawasaki, Takafumi Kitazawa

**Affiliations:** a Department of Chemistry, Faculty of Science, Toho University, 2-2-1 Miyama, Funabashi, Chiba 274-8510, Japan; b Department of Chemistry, Faculty of Science, Toho University, 2-2-1 Miyama, Funabashi, Chiba 274-8510, Japan; c Research Center for Materials with Integrated Properties, Toho University, Miyama, Funabashi, Chiba 274-8510, Japan; University of Hyogo, Japan

**Keywords:** crystal structure, uranium(VI) complex, hemisolvate, uran­yl(VI) ion, hydrogen bond

## Abstract

Uran­yl(VI) complexes have been researched in many laboratories due to their relevance to actinide coordination chemistry and nuclear fuel reprocessing. The asymmetric unit of the title uranyl complex, [U(C_5_H_7_O_2_)_2_O_2_(C_5_H_4_Cl_2_N_2_)]·0.5CH_3_OH or [UO_2_(acac)_2_(4-amino-3,5-di­chloro­pyridine)]·0.5CH_3_OH, contains two crystallographically independent uran­yl(VI) complex mol­ecules and one methanol solvent mol­ecule. Each U^VI^ atom adopts a UNO_6_ penta­gonal–bipyramidal coordination geometry, in which two uranyl O atoms occupy the axial positions, whereas four O atoms from two chelating bidentate acac ligands and one N atom from a 4-amino-3,5-di­chloro­pyridine ligand define the equatorial plane. The U=O bond lengths are 1.757 (10) and 1.807 (11) Å for one independent mol­ecule and 1.752 (11) and 1.766 (11) Å for the other, while the U=O(acac) and U—N bond lengths lie in the ranges 2.302 (11)–2.384 (10) Å and 2.591 (13)–2.645 (13) Å, respectively. The bond lengths around the U atoms decrease in the order U—N > U—Oacac > U=O. The uranyl units are slightly bent, with O=U=O angles of 177.0 (5) and 176.5 (5)°. In the crystal, the methanol solvent mol­ecule is linked to the amino group in the coordinated ligand by an inter­molecular N—H⋯O hydrogen bond [N⋯O = 2.90 (2) Å], which is likely to contribute to the low-symmetry crystal arrangement.

## Abstract 2

The asymmetric unit of the title uranyl complex, [U(C_5_H_7_O_2_)_2_O_2_(C_5_H_4_Cl_2_N_2_)]·0.5CH_3_OH or [UO_2_(acac)_2_(4-amino-3,5-di­chloro­pyridine)]·0.5CH_3_OH, contains two crystallographically independent uran­yl(VI) complex mol­ecules and one methanol solvent mol­ecule. Each U^VI^ atom adopts a UNO_6_ penta­gonal–bipyramidal coordination geometry, in which two uranyl O atoms occupy the axial positions, whereas four O atoms from two chelating bidentate acac ligands and one N atom from a 4-amino-3,5-di­chloro­pyridine ligand define the equatorial plane. In the crystal, the methanol solvent mol­ecule is linked to the amino group in the coordinated ligand by an inter­molecular N—H⋯O hydrogen bond, which is likely to contribute to the low-symmetry crystal arrangement.

## 1. Chemical context

Uranyl(VI) complexes have been researched in many laboratories (Alcock *et al.*, 1984 ▸; Kawasaki *et al.*, 2006 ▸; Kawasaki & Kitazawa, 2008*a* ▸,*b* ▸, 2015 ▸; Kelly *et al.*, 2025 ▸) due to their relevance to actinide coordination chemistry and nuclear fuel reprocessing. Among the various ligand systems employed for uran­yl(VI), β-diketonate ligands have been extensively studied, and acetyl­acetonate (acac) is one of the simplest and most important ligands in this class. A number of uran­yl(VI) acetyl­acetonate complexes containing additional N-donor ligands have been structurally characterized. In this context, we report here the synthesis and crystal structure of a new uran­yl(VI) acetyl­acetonate complex [UO_2_(acac)_2_(4-amino-3,5-di­chloro­pyrid­ine)]·0.5CH_3_OH, which contains 4-amino-3,5-di­chloro­pyridine as a neutral N-donor ligand.

## 2. Structural commentary

The crystal structure of the synthesized complex (Fig. 1 ▸) contains two crystallographically independent [UO_2_(acac)_2_(4-amino-3,5-di­chloro­pyridine)] mol­ecules and one methanol mol­ecule in the asymmetric unit. In each of the two crystallographically independent complexes, the uranium(VI) atom is seven-coordinated and adopts a pentagonal–bipyramidal geometry. The axial positions are occupied by the two uranyl O atoms, whereas the equatorial plane comprises four O atoms from two chelating acetylacetonate (acac) ligands and the pyridyl N atom of a coordinated 4-amino-3,5-dichloropyridine ligand.
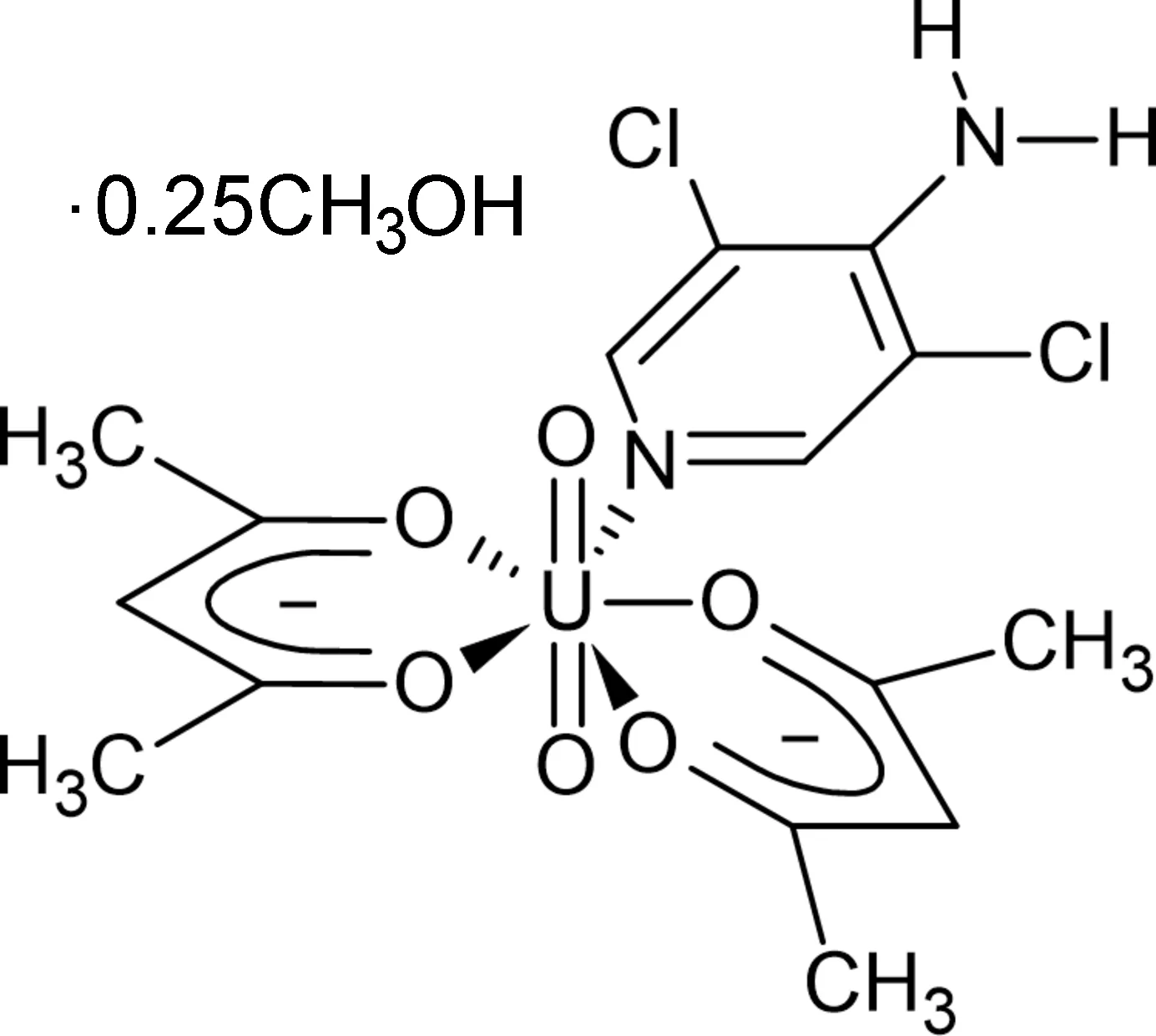


For one independent mol­ecule, the axial bond lengths are U1—O5 = 1.757 (10) Å and U1—O6 = 1.807 (11) Å, whereas for the second they are U2—O12 = 1.752 (11) Å and U2—O11 = 1.766 (11) Å. The equatorial U—O distances are in the range 2.302 (11)–2.384 (10) Å, while the U—N distances are 2.645 (13) and 2.591 (13) Å. As in related uran­yl(VI) acetyl­acetonate complexes (Kawasaki *et al.*, 2006 ▸; Kawasaki & Kitazawa, 2008*a* ▸,*b* ▸, 2015 ▸), the bond lengths decrease in the order U—N > U—Oacac > U=O.

The uranyl groups are slightly bent, with O5—U1—O6 = 170.0 (5)° and O12—U2—O11 = 176.5 (5)°. The crystal belongs to the triclinic space group *P*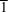 with *Z* = 4. The asymmetric unit contains two crystallographically independent complex molecules and one methanol molecule, corresponding to *Z*′ = 2. The two independent complexes exhibit slight differences in their local coordination geometries.

## 3. Supra­molecular features

A methanol solvent mol­ecule is incorporated into the crystal and is linked to theamino group of the coordinated ligand through an inter­molecular N4—H4*B*⋯O13 hydrogen bond (Table 1 ▸). This interaction likely helps to consolidate the crystal structure. The presence of methanol may also contribute to the low-symmetry crystal arrangement, even though 4-amino-3,5-di­chloro­pyridine has same symmetry as unsubstituted pyridine (*C*_2*v*_). The inclusion of the methanol mol­ecule distinguishes the title compound from related solvent-free uran­yl(VI) acetyl­acetonate complexes and may contribute to the slight differences observed between the axial U=O bond lengths. In contrast, [UO_2_(acac)_2_(pyridine)] (Alcock *et al.*, 1984), whose pyridine ligand cannot form analogous N—H⋯O hydrogen bonds, crystallizes without solvent in the higher-symmetry space group *Fdd*2.

## 4. Synthesis and crystallization

The title complex was synthesized by a direct reaction of acetyl­acetone, 4-amino-3,5-di­chloro­pyridine, and uranyl nitrate hexa­hydrate in methanol. Acetyl­acetone (0.953 g, 0.95 mmol) and 4-amino-3,5-di­chloro­pyridine (0.1618 g, 1 mmol) were dissolved in 10 ml of methanol, and uranyl nitrate hexa­hydrate (0.15 g, 0.3 mmol) was then added to the resulting solution. The mixture was left to stand in a fume hood for two weeks. Orange single crystals suitable for X-ray diffraction analysis were obtained by slow evaporation of the solvent.

## 5. Refinement

Crystal data, data collection and structure refinement details are summarized in Table 2 ▸. H atoms were placed in calculated positions (O—H = 0.82, N—H = 0.86, C—H = 0.93–0.96 Å) and refined as riding with *U*_i__so_(H) = 1.2–1.5*U*_eq_ of the parent atom.

## Supplementary Material

Crystal structure: contains datablock(s) I. DOI: 10.1107/S2056989026009618/ox2025sup1.cif

Structure factors: contains datablock(s) I. DOI: 10.1107/S2056989026009618/ox2025Isup2.hkl

CCDC reference: 2588059

Additional supporting information:  crystallographic information; 3D view; checkCIF report

## Figures and Tables

**Figure 1:**
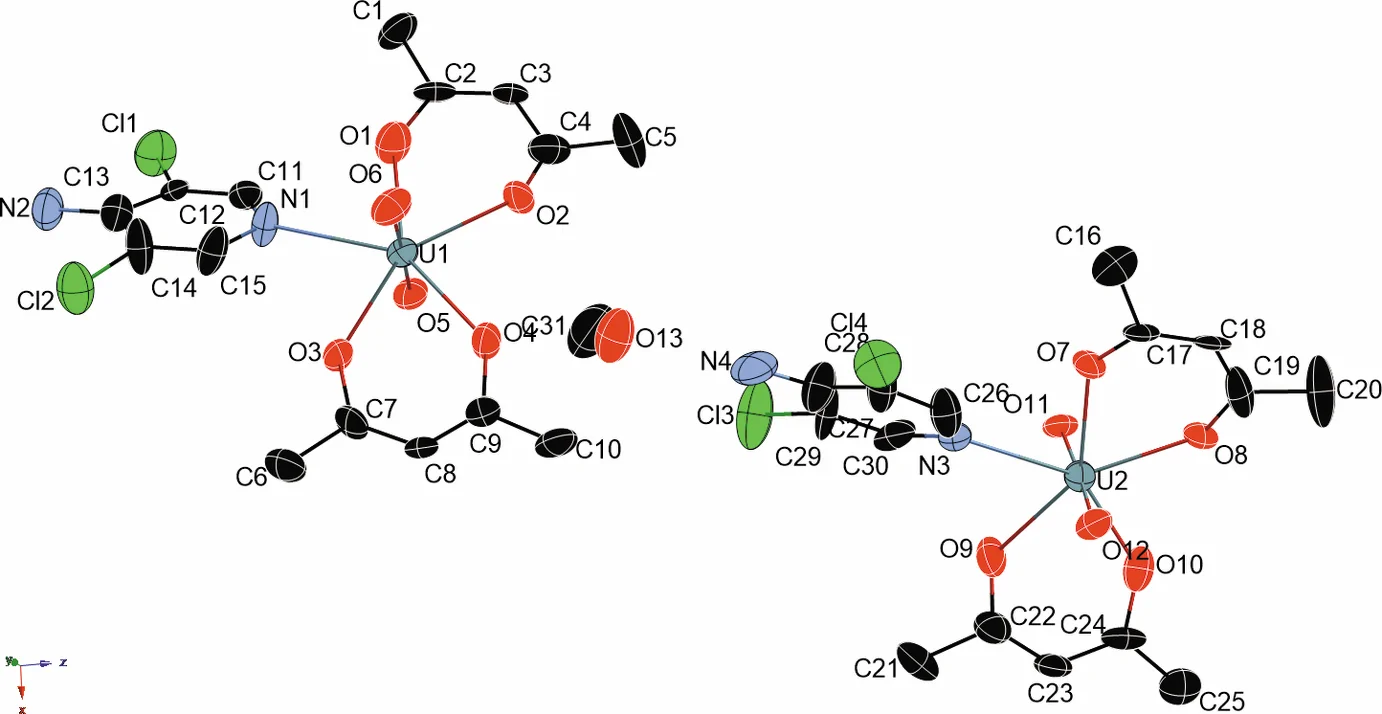
Ellipsoid plot of the title compound with displacement ellipsoids drawn at the 50% probability level.

**Table 1:** Hydrogen-bond geometry (Å, °)

*D*—H⋯*A*	*D*—H	H⋯*A*	*D*⋯*A*	*D*—H⋯*A*
N4—H4*B*⋯O13	0.86	2.09	2.90 (2)	157

**Table 2:** Experimental details

Crystal data	
Chemical formula	[U(C_5_H_7_O_2_)_2_O_2_(C_5_H_4_Cl_2_N_2_)]·0.5CH_4_O
*M*_r_	647.26
Crystal system, space group	Triclinic, *P* 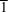
Temperature (K)	273
*a*, *b*, *c* (Å)	8.1200 (13), 11.7640 (19), 22.830 (4)
α, β, γ (°)	97.657 (3), 91.181 (3), 97.175 (3)
*V* (Å^3^)	2142.9 (6)
*Z*	4
Radiation type	Mo *K*α
μ (mm^−1^)	7.86
Crystal size (mm)	0.30 × 0.05 × 0.05

Data collection	
Diffractometer	Bruker APEXII CCD
Absorption correction	Multi-scan (*SADABS*; Krause *et al.*, 2015 ▸)
*T*_min_, *T*_max_	0.453, 0.745
No. of measured, independent and observed [*I* > 2σ(*I*)] reflections	11317, 7468, 4722
*R*_int_	0.052
(sin θ/λ)_max_ (Å^−1^)	0.596

Refinement	
*R*[*F*^2^ > 2σ(*F*^2^)], *wR*(*F*^2^), *S*	0.057, 0.178, 1.01
No. of reflections	7468
No. of parameters	494
H-atom treatment	H-atom parameters constrained
Δρ_max_, Δρ_min_ (e Å^−3^)	1.29, −1.59

Computer programs: *APEX2* and *SAINT* (Bruker, 2025 ▸), *SHELXT2014/5* (Sheldrick, 2015*a* ▸), *SHELXL2019/2* (Sheldrick, 2015*b* ▸) and *SHELXTL* (Sheldrick, 2008 ▸).

## supplementary crystallographic information

### (4-Amino-3,5-dichloropyridine-κN1)dioxidobis(pentane-2,4-dionato-κ2O,O')uranium(VI) methanol hemisolvate Crystal data

**Table:**

[U(C_5_H_7_O_2_)_2_O_2_(C_5_H_4_Cl_2_N_2_)]·0.5CH_4_O	*Z* = 4
*M**_r_* = 647.26	*F*(000) = 1220
Triclinic, *P*1	*D*_x_ = 2.006 Mg m^−^^3^
*a* = 8.1200 (13) Å	Mo *K*α radiation, λ = 0.71073 Å
*b* = 11.7640 (19) Å	Cell parameters from 1862 reflections
*c* = 22.830 (4) Å	θ = 2.3–21.4°
α = 97.657 (3)°	µ = 7.86 mm^−^^1^
β = 91.181 (3)°	*T* = 273 K
γ = 97.175 (3)°	Needle, orange
*V* = 2142.9 (6) Å^3^	0.30 × 0.05 × 0.05 mm

### (4-Amino-3,5-dichloropyridine-κN1)dioxidobis(pentane-2,4-dionato-κ2O,O')uranium(VI) methanol hemisolvate Data collection

**Table:**

Bruker APEXII CCD diffractometer	4722 reflections with *I* > 2σ(*I*)
Detector resolution: 8.336 pixels mm^-1^	*R*_int_ = 0.052
φ and ω scans	θ_max_ = 25.1°, θ_min_ = 0.9°
Absorption correction: multi-scan (SADABS; Krause *et al.*, 2015)	*h* = −9→9
*T*_min_ = 0.453, *T*_max_ = 0.745	*k* = −14→13
11317 measured reflections	*l* = −27→24
7468 independent reflections	

### (4-Amino-3,5-dichloropyridine-κN1)dioxidobis(pentane-2,4-dionato-κ2O,O')uranium(VI) methanol hemisolvate Refinement

**Table:**

Refinement on *F*^2^	0 restraints
Least-squares matrix: full	Hydrogen site location: inferred from neighbouring sites
*R*[*F*^2^ > 2σ(*F*^2^)] = 0.057	H-atom parameters constrained
*wR*(*F*^2^) = 0.178	*w* = 1/[σ^2^(*F*_o_^2^) + (0.0894*P*)^2^ + 2.9867*P*] where *P* = (*F*_o_^2^ + 2*F*_c_^2^)/3
*S* = 1.01	(Δ/σ)_max_ = 0.001
7468 reflections	Δρ_max_ = 1.29 e Å^−^^3^
494 parameters	Δρ_min_ = −1.59 e Å^−^^3^

### (4-Amino-3,5-dichloropyridine-κN1)dioxidobis(pentane-2,4-dionato-κ2O,O')uranium(VI) methanol hemisolvate Special details

**Table:**

Geometry. All esds (except the esd in the dihedral angle between two l.s. planes) are estimated using the full covariance matrix. The cell esds are taken into account individually in the estimation of esds in distances, angles and torsion angles; correlations between esds in cell parameters are only used when they are defined by crystal symmetry. An approximate (isotropic) treatment of cell esds is used for estimating esds involving l.s. planes.
Refinement. The crystal structure was determined via single-crystal X-ray diffraction (XRD) using a Bruker SMART diffractometer with a Mo-Kα line at 296 K. This structure was clarified using the SHELX 2014 software. [Sheldrick, G. M. (2008), Sheldrick, G. M. (2015).] The details of crystal paramater are as follows: triclinic, P-1, a = 8.1200 (13) Å, b = 11.7640 (19) Å, c = 22.830 (4) Å, α = 97.657 (3)°, β = 91.181 (3)°, γ = 97.175 (3)°, V = 2142.9 (6) Å3, Z = 4. Refinement of the structure converged at R1 = 0.0582 and wR2 = 0.1459 for reflections with I > 2σ(I), and R1 = 0.1047 and wR2 = 0.1860 for all data.

### (4-Amino-3,5-dichloropyridine-κN1)dioxidobis(pentane-2,4-dionato-κ2O,O')uranium(VI) methanol hemisolvate Fractional atomic coordinates and isotropic or equivalent isotropic displacement parameters (Å^2^)

**Table:**

	*x*	*y*	*z*	*U*_iso_*/*U*_eq_	
U1	−0.07597 (7)	0.20276 (5)	0.13559 (3)	0.0344 (2)	
U2	0.59276 (7)	0.26754 (5)	0.67937 (3)	0.0362 (2)	
Cl1	−0.2601 (6)	0.5665 (4)	−0.0125 (2)	0.0618 (13)	
Cl2	−0.0728 (7)	0.1800 (4)	−0.1341 (2)	0.0664 (14)	
Cl3	0.4104 (9)	0.3717 (5)	0.4330 (2)	0.091 (2)	
Cl4	0.2105 (7)	−0.0625 (4)	0.4799 (2)	0.0719 (15)	
O1	−0.2999 (16)	0.3063 (10)	0.1474 (5)	0.058 (3)	
O2	−0.1775 (14)	0.1962 (11)	0.2320 (5)	0.054 (3)	
O3	0.1270 (15)	0.1464 (13)	0.0704 (5)	0.069 (4)	
O4	0.1182 (14)	0.1139 (10)	0.1872 (5)	0.049 (3)	
O5	0.0528 (12)	0.3340 (9)	0.1565 (5)	0.045 (3)	
O6	−0.2162 (13)	0.0719 (10)	0.1124 (5)	0.054 (3)	
O7	0.3173 (12)	0.1706 (12)	0.6809 (5)	0.057 (3)	
O8	0.5240 (12)	0.2543 (11)	0.7777 (5)	0.050 (3)	
O9	0.7737 (15)	0.3357 (11)	0.6119 (5)	0.058 (3)	
O10	0.8358 (15)	0.3493 (10)	0.7321 (5)	0.054 (3)	
O11	0.5097 (12)	0.4000 (10)	0.6833 (5)	0.045 (3)	
O12	0.6697 (13)	0.1339 (9)	0.6711 (5)	0.045 (3)	
O13	0.1712 (19)	0.2559 (12)	0.3094 (6)	0.075 (4)	
H13A	0.183582	0.220844	0.276568	0.112*	
N1	−0.1511 (18)	0.2761 (12)	0.0350 (6)	0.048 (4)	
N2	−0.1963 (18)	0.4158 (13)	−0.1220 (6)	0.055 (4)	
H2A	−0.223235	0.484251	−0.121929	0.066*	
H2B	−0.178388	0.375064	−0.154870	0.066*	
N3	0.4616 (15)	0.2121 (12)	0.5734 (6)	0.039 (3)	
N4	0.2409 (17)	0.1239 (15)	0.4037 (7)	0.069 (5)	
H4A	0.192622	0.054843	0.392658	0.082*	
H4B	0.247552	0.174049	0.379433	0.082*	
C1	−0.522 (2)	0.4171 (17)	0.1714 (8)	0.061 (6)	
H1A	−0.479718	0.458898	0.140364	0.091*	
H1B	−0.542183	0.470803	0.205143	0.091*	
H1C	−0.623226	0.369133	0.157891	0.091*	
C2	−0.3953 (18)	0.3424 (15)	0.1885 (8)	0.045 (4)	
C3	−0.3821 (19)	0.3159 (17)	0.2462 (8)	0.053 (5)	
H3	−0.454533	0.345263	0.273633	0.063*	
C4	−0.266 (2)	0.2474 (16)	0.2658 (9)	0.055 (5)	
C5	−0.277 (3)	0.218 (2)	0.3272 (8)	0.090 (8)	
H5A	−0.239528	0.144113	0.328599	0.135*	
H5B	−0.389715	0.215231	0.339026	0.135*	
H5C	−0.207743	0.276005	0.353594	0.135*	
C6	0.360 (2)	0.1290 (19)	0.0196 (9)	0.068 (6)	
H6A	0.311161	0.064752	−0.008436	0.101*	
H6B	0.475550	0.123918	0.025816	0.101*	
H6C	0.346675	0.199962	0.004630	0.101*	
C7	0.277 (2)	0.1264 (16)	0.0753 (7)	0.049 (5)	
C8	0.3410 (18)	0.0973 (14)	0.1267 (7)	0.041 (4)	
H8	0.450623	0.082188	0.125337	0.049*	
C9	0.2709 (19)	0.0878 (13)	0.1771 (7)	0.037 (4)	
C10	0.346 (2)	0.0437 (16)	0.2290 (8)	0.055 (5)	
H10A	0.464783	0.056066	0.227780	0.082*	
H10B	0.309247	−0.037543	0.227428	0.082*	
H10C	0.311422	0.084274	0.265077	0.082*	
C11	−0.1928 (18)	0.3837 (14)	0.0349 (7)	0.039 (4)	
H11	−0.211588	0.427538	0.070574	0.047*	
C12	−0.2076 (16)	0.4291 (13)	−0.0164 (7)	0.033 (4)	
C13	−0.183 (2)	0.3734 (14)	−0.0718 (7)	0.045 (4)	
C14	−0.132 (3)	0.2619 (15)	−0.0694 (7)	0.062 (6)	
C15	−0.117 (2)	0.2199 (15)	−0.0160 (8)	0.057 (5)	
H15	−0.080721	0.147893	−0.016260	0.068*	
C16	0.070 (2)	0.0524 (14)	0.6920 (9)	0.058 (5)	
H16A	−0.004761	0.107794	0.687329	0.087*	
H16B	0.024962	0.000684	0.718434	0.087*	
H16C	0.085255	0.009033	0.654175	0.087*	
C17	0.2423 (17)	0.1172 (13)	0.7180 (8)	0.036 (4)	
C18	0.2822 (17)	0.1227 (15)	0.7755 (8)	0.042 (4)	
H18	0.214904	0.076400	0.797827	0.051*	
C19	0.418 (3)	0.1935 (16)	0.8040 (8)	0.060 (6)	
C20	0.437 (4)	0.2045 (19)	0.8711 (9)	0.100 (9)	
H20A	0.330356	0.188052	0.887446	0.150*	
H20B	0.485116	0.281822	0.886510	0.150*	
H20C	0.508652	0.150696	0.881731	0.150*	
C21	1.002 (3)	0.370 (2)	0.5507 (9)	0.086 (7)	
H21A	0.949076	0.307644	0.522452	0.129*	
H21B	1.120097	0.368481	0.551083	0.129*	
H21C	0.977506	0.442287	0.539904	0.129*	
C22	0.939 (2)	0.3563 (15)	0.6115 (8)	0.051 (5)	
C23	1.0416 (19)	0.3714 (15)	0.6603 (8)	0.047 (4)	
H23	1.154888	0.383396	0.654058	0.056*	
C24	0.9958 (18)	0.3710 (13)	0.7187 (9)	0.045 (4)	
C25	1.126 (2)	0.4073 (15)	0.7663 (8)	0.055 (5)	
H25A	1.227502	0.379869	0.753856	0.082*	
H25B	1.090938	0.375416	0.801336	0.082*	
H25C	1.142609	0.490181	0.774666	0.082*	
C26	0.378 (2)	0.1069 (15)	0.5540 (8)	0.056 (5)	
H26	0.374501	0.051136	0.579440	0.068*	
C27	0.299 (3)	0.0761 (16)	0.4998 (8)	0.057 (5)	
C28	0.305 (3)	0.1532 (15)	0.4585 (8)	0.059 (5)	
C29	0.395 (3)	0.2648 (16)	0.4781 (8)	0.062 (6)	
C30	0.4663 (19)	0.2908 (16)	0.5347 (8)	0.050 (5)	
H30	0.519497	0.365208	0.546750	0.060*	
C31	0.187 (3)	0.376 (2)	0.3062 (11)	0.107 (9)	
H31A	0.093427	0.393203	0.284540	0.161*	
H31B	0.287289	0.397940	0.286492	0.161*	
H31C	0.192820	0.417418	0.345460	0.161*	

### (4-Amino-3,5-dichloropyridine-κN1)dioxidobis(pentane-2,4-dionato-κ2O,O')uranium(VI) methanol hemisolvate Atomic displacement parameters (Å^2^)

**Table:**

	*U*^11^	*U*^22^	*U*^33^	*U*^12^	*U*^13^	*U*^23^
U1	0.0314 (3)	0.0383 (4)	0.0362 (4)	0.0095 (3)	0.0013 (3)	0.0097 (3)
U2	0.0330 (3)	0.0411 (4)	0.0338 (4)	0.0015 (3)	0.0011 (3)	0.0055 (3)
Cl1	0.080 (3)	0.044 (3)	0.066 (3)	0.021 (2)	−0.001 (3)	0.013 (2)
Cl2	0.101 (4)	0.053 (3)	0.046 (3)	0.017 (3)	0.006 (3)	0.003 (2)
Cl3	0.163 (6)	0.054 (3)	0.054 (3)	0.000 (4)	−0.026 (4)	0.015 (3)
Cl4	0.076 (3)	0.057 (3)	0.074 (4)	−0.013 (3)	−0.002 (3)	−0.002 (3)
O1	0.072 (9)	0.052 (8)	0.054 (8)	0.019 (7)	−0.003 (7)	0.015 (6)
O2	0.049 (7)	0.082 (9)	0.046 (7)	0.037 (7)	0.020 (6)	0.029 (7)
O3	0.062 (8)	0.123 (12)	0.044 (8)	0.065 (9)	0.012 (6)	0.034 (8)
O4	0.058 (8)	0.061 (8)	0.036 (7)	0.027 (6)	0.005 (6)	0.013 (6)
O5	0.032 (6)	0.052 (7)	0.049 (7)	−0.006 (5)	−0.006 (5)	0.012 (6)
O6	0.042 (7)	0.052 (8)	0.068 (9)	−0.003 (6)	−0.016 (6)	0.015 (6)
O7	0.023 (6)	0.104 (10)	0.034 (7)	−0.016 (6)	0.002 (5)	−0.001 (6)
O8	0.023 (6)	0.078 (9)	0.046 (7)	−0.008 (6)	0.006 (5)	0.011 (6)
O9	0.050 (8)	0.082 (9)	0.037 (7)	−0.017 (7)	−0.001 (6)	0.018 (6)
O10	0.075 (9)	0.052 (8)	0.034 (7)	0.005 (7)	−0.011 (6)	−0.001 (5)
O11	0.025 (5)	0.068 (8)	0.044 (7)	0.019 (5)	0.001 (5)	0.000 (6)
O12	0.036 (6)	0.049 (7)	0.049 (7)	0.009 (5)	−0.007 (5)	0.000 (5)
O13	0.111 (12)	0.059 (9)	0.054 (9)	0.025 (9)	−0.017 (8)	−0.004 (7)
N1	0.072 (10)	0.046 (9)	0.027 (8)	0.014 (8)	−0.008 (7)	0.008 (6)
N2	0.067 (10)	0.064 (10)	0.039 (9)	0.020 (8)	0.000 (8)	0.017 (8)
N3	0.027 (7)	0.051 (9)	0.040 (8)	0.000 (6)	−0.001 (6)	0.010 (7)
N4	0.037 (9)	0.091 (13)	0.068 (12)	−0.006 (8)	−0.014 (8)	−0.011 (10)
C1	0.051 (11)	0.080 (14)	0.055 (12)	0.034 (11)	−0.016 (9)	−0.001 (10)
C2	0.021 (8)	0.050 (11)	0.068 (13)	0.018 (7)	0.007 (8)	0.005 (9)
C3	0.027 (9)	0.089 (14)	0.049 (12)	0.032 (9)	0.012 (8)	0.011 (10)
C4	0.038 (10)	0.057 (12)	0.073 (14)	0.012 (9)	0.009 (10)	0.016 (10)
C5	0.12 (2)	0.11 (2)	0.041 (13)	0.015 (17)	0.031 (13)	0.020 (13)
C6	0.048 (11)	0.094 (16)	0.069 (14)	0.025 (11)	0.022 (10)	0.025 (12)
C7	0.051 (11)	0.064 (12)	0.038 (10)	0.028 (9)	0.020 (8)	0.003 (9)
C8	0.019 (8)	0.059 (11)	0.048 (11)	0.009 (7)	0.001 (7)	0.013 (8)
C9	0.036 (9)	0.028 (8)	0.044 (10)	0.006 (7)	−0.001 (8)	−0.001 (7)
C10	0.036 (10)	0.065 (13)	0.065 (13)	0.022 (9)	−0.006 (9)	−0.001 (10)
C11	0.035 (9)	0.041 (10)	0.044 (10)	0.012 (7)	0.003 (7)	0.007 (8)
C12	0.019 (7)	0.048 (10)	0.036 (9)	0.009 (7)	−0.002 (6)	0.014 (7)
C13	0.052 (11)	0.042 (10)	0.042 (11)	−0.001 (8)	−0.004 (8)	0.008 (8)
C14	0.122 (18)	0.038 (10)	0.028 (10)	0.014 (11)	0.005 (11)	0.003 (8)
C15	0.094 (15)	0.036 (10)	0.043 (11)	0.020 (10)	−0.018 (10)	0.008 (8)
C16	0.055 (11)	0.031 (10)	0.084 (15)	−0.012 (8)	−0.014 (10)	0.013 (9)
C17	0.013 (7)	0.042 (9)	0.055 (11)	−0.001 (6)	0.006 (7)	0.014 (8)
C18	0.009 (7)	0.062 (12)	0.055 (12)	0.003 (7)	0.009 (7)	0.007 (9)
C19	0.114 (18)	0.043 (11)	0.036 (10)	0.044 (12)	0.025 (11)	0.019 (8)
C20	0.19 (3)	0.068 (15)	0.040 (13)	0.004 (17)	0.005 (15)	0.027 (11)
C21	0.061 (13)	0.14 (2)	0.064 (14)	0.007 (14)	0.031 (11)	0.021 (14)
C22	0.046 (11)	0.052 (11)	0.060 (13)	0.002 (9)	0.011 (9)	0.024 (9)
C23	0.023 (8)	0.059 (12)	0.063 (13)	0.003 (8)	0.013 (8)	0.025 (9)
C24	0.022 (8)	0.034 (9)	0.083 (14)	0.008 (7)	0.011 (9)	0.016 (9)
C25	0.053 (11)	0.044 (11)	0.070 (13)	0.013 (9)	0.003 (10)	0.011 (9)
C26	0.095 (15)	0.037 (10)	0.039 (11)	0.014 (10)	0.008 (10)	0.008 (8)
C27	0.087 (14)	0.053 (12)	0.031 (10)	0.018 (11)	−0.003 (10)	−0.001 (9)
C28	0.087 (15)	0.038 (10)	0.045 (11)	−0.006 (10)	−0.025 (11)	−0.001 (9)
C29	0.107 (17)	0.051 (12)	0.030 (10)	0.019 (11)	−0.022 (10)	0.014 (8)
C30	0.028 (9)	0.055 (11)	0.062 (13)	0.009 (8)	−0.006 (8)	−0.011 (10)
C31	0.12 (2)	0.12 (2)	0.09 (2)	0.037 (19)	−0.027 (17)	0.015 (17)

### (4-Amino-3,5-dichloropyridine-κN1)dioxidobis(pentane-2,4-dionato-κ2O,O')uranium(VI) methanol hemisolvate Geometric parameters (Å, º)

**Table:**

U1—O5	1.757 (10)	C6—C7	1.45 (2)
U1—O6	1.807 (11)	C6—H6A	0.9600
U1—O1	2.313 (12)	C6—H6B	0.9600
U1—O3	2.341 (11)	C6—H6C	0.9600
U1—O4	2.373 (10)	C7—C8	1.37 (2)
U1—O2	2.373 (11)	C8—C9	1.31 (2)
U1—N1	2.645 (13)	C8—H8	0.9300
U2—O12	1.752 (11)	C9—C10	1.50 (2)
U2—O11	1.766 (11)	C10—H10A	0.9600
U2—O9	2.302 (11)	C10—H10B	0.9600
U2—O10	2.321 (11)	C10—H10C	0.9600
U2—O8	2.346 (11)	C11—C12	1.36 (2)
U2—O7	2.384 (10)	C11—H11	0.9300
U2—N3	2.591 (13)	C12—C13	1.38 (2)
Cl1—C12	1.713 (15)	C13—C14	1.43 (2)
Cl2—C14	1.762 (18)	C14—C15	1.38 (2)
Cl3—C29	1.723 (18)	C15—H15	0.9300
Cl4—C27	1.696 (19)	C16—C17	1.57 (2)
O1—C2	1.292 (19)	C16—H16A	0.9600
O2—C4	1.22 (2)	C16—H16B	0.9600
O3—C7	1.272 (19)	C16—H16C	0.9600
O4—C9	1.332 (17)	C17—C18	1.34 (2)
O7—C17	1.245 (18)	C18—C19	1.39 (3)
O8—C19	1.26 (2)	C18—H18	0.9300
O9—C22	1.33 (2)	C19—C20	1.52 (2)
O10—C24	1.341 (18)	C20—H20A	0.9600
O13—C31	1.41 (3)	C20—H20B	0.9600
O13—H13A	0.8200	C20—H20C	0.9600
N1—C15	1.31 (2)	C21—C22	1.51 (2)
N1—C11	1.351 (19)	C21—H21A	0.9600
N2—C13	1.32 (2)	C21—H21B	0.9600
N2—H2A	0.8600	C21—H21C	0.9600
N2—H2B	0.8600	C22—C23	1.36 (2)
N3—C26	1.35 (2)	C23—C24	1.39 (2)
N3—C30	1.36 (2)	C23—H23	0.9300
N4—C28	1.33 (2)	C24—C25	1.48 (2)
N4—H4A	0.8600	C25—H25A	0.9600
N4—H4B	0.8600	C25—H25B	0.9600
C1—C2	1.51 (2)	C25—H25C	0.9600
C1—H1A	0.9600	C26—C27	1.37 (2)
C1—H1B	0.9600	C26—H26	0.9300
C1—H1C	0.9600	C27—C28	1.39 (2)
C2—C3	1.40 (2)	C28—C29	1.43 (2)
C3—C4	1.42 (2)	C29—C30	1.38 (2)
C3—H3	0.9300	C30—H30	0.9300
C4—C5	1.49 (3)	C31—H31A	0.9600
C5—H5A	0.9600	C31—H31B	0.9600
C5—H5B	0.9600	C31—H31C	0.9600
C5—H5C	0.9600		
			
O5—U1—O6	177.0 (5)	C8—C7—C6	126.5 (15)
O5—U1—O1	87.5 (5)	C9—C8—C7	129.6 (15)
O6—U1—O1	90.0 (5)	C9—C8—H8	115.2
O5—U1—O3	88.3 (5)	C7—C8—H8	115.2
O6—U1—O3	92.9 (5)	C8—C9—O4	122.4 (15)
O1—U1—O3	144.5 (4)	C8—C9—C10	125.7 (14)
O5—U1—O4	87.1 (4)	O4—C9—C10	111.9 (14)
O6—U1—O4	95.9 (5)	C9—C10—H10A	109.5
O1—U1—O4	143.6 (4)	C9—C10—H10B	109.5
O3—U1—O4	71.2 (4)	H10A—C10—H10B	109.5
O5—U1—O2	93.4 (5)	C9—C10—H10C	109.5
O6—U1—O2	87.2 (5)	H10A—C10—H10C	109.5
O1—U1—O2	70.4 (4)	H10B—C10—H10C	109.5
O3—U1—O2	145.1 (4)	N1—C11—C12	121.1 (15)
O4—U1—O2	74.0 (4)	N1—C11—H11	119.4
O5—U1—N1	89.1 (5)	C12—C11—H11	119.4
O6—U1—N1	88.6 (5)	C11—C12—C13	125.0 (15)
O1—U1—N1	70.0 (4)	C11—C12—Cl1	118.1 (12)
O3—U1—N1	74.6 (4)	C13—C12—Cl1	116.9 (12)
O4—U1—N1	145.7 (4)	N2—C13—C12	125.9 (16)
O2—U1—N1	140.2 (4)	N2—C13—C14	122.1 (16)
O12—U2—O11	176.5 (5)	C12—C13—C14	111.9 (15)
O12—U2—O9	91.5 (5)	C15—C14—C13	120.9 (16)
O11—U2—O9	87.4 (5)	C15—C14—Cl2	118.7 (14)
O12—U2—O10	88.9 (4)	C13—C14—Cl2	120.2 (13)
O11—U2—O10	93.8 (5)	N1—C15—C14	123.6 (16)
O9—U2—O10	73.2 (4)	N1—C15—H15	118.2
O12—U2—O8	92.6 (5)	C14—C15—H15	118.2
O11—U2—O8	90.0 (5)	C17—C16—H16A	109.5
O9—U2—O8	150.0 (4)	C17—C16—H16B	109.5
O10—U2—O8	77.1 (4)	H16A—C16—H16B	109.5
O12—U2—O7	89.9 (5)	C17—C16—H16C	109.5
O11—U2—O7	88.8 (5)	H16A—C16—H16C	109.5
O9—U2—O7	138.9 (4)	H16B—C16—H16C	109.5
O10—U2—O7	147.9 (4)	O7—C17—C18	127.7 (15)
O8—U2—O7	70.9 (4)	O7—C17—C16	111.4 (15)
O12—U2—N3	88.0 (5)	C18—C17—C16	120.3 (15)
O11—U2—N3	88.6 (5)	C17—C18—C19	123.9 (16)
O9—U2—N3	70.1 (4)	C17—C18—H18	118.1
O10—U2—N3	143.1 (4)	C19—C18—H18	118.1
O8—U2—N3	139.7 (4)	O8—C19—C18	123.8 (17)
O7—U2—N3	68.8 (4)	O8—C19—C20	117 (2)
C2—O1—U1	139.3 (11)	C18—C19—C20	118.9 (19)
C4—O2—U1	138.9 (12)	C19—C20—H20A	109.5
C7—O3—U1	135.9 (11)	C19—C20—H20B	109.5
C9—O4—U1	134.6 (10)	H20A—C20—H20B	109.5
C17—O7—U2	132.9 (10)	C19—C20—H20C	109.5
C19—O8—U2	136.4 (12)	H20A—C20—H20C	109.5
C22—O9—U2	133.1 (11)	H20B—C20—H20C	109.5
C24—O10—U2	135.0 (11)	C22—C21—H21A	109.5
C31—O13—H13A	109.5	C22—C21—H21B	109.5
C15—N1—C11	117.3 (14)	H21A—C21—H21B	109.5
C15—N1—U1	120.9 (11)	C22—C21—H21C	109.5
C11—N1—U1	120.4 (10)	H21A—C21—H21C	109.5
C13—N2—H2A	120.0	H21B—C21—H21C	109.5
C13—N2—H2B	120.0	O9—C22—C23	124.6 (16)
H2A—N2—H2B	120.0	O9—C22—C21	113.0 (16)
C26—N3—C30	115.9 (14)	C23—C22—C21	122.3 (16)
C26—N3—U2	122.9 (11)	C22—C23—C24	127.0 (15)
C30—N3—U2	121.2 (11)	C22—C23—H23	116.5
C28—N4—H4A	120.0	C24—C23—H23	116.5
C28—N4—H4B	120.0	O10—C24—C23	121.0 (16)
H4A—N4—H4B	120.0	O10—C24—C25	120.3 (16)
C2—C1—H1A	109.5	C23—C24—C25	118.4 (15)
C2—C1—H1B	109.5	C24—C25—H25A	109.5
H1A—C1—H1B	109.5	C24—C25—H25B	109.5
C2—C1—H1C	109.5	H25A—C25—H25B	109.5
H1A—C1—H1C	109.5	C24—C25—H25C	109.5
H1B—C1—H1C	109.5	H25A—C25—H25C	109.5
O1—C2—C3	122.5 (14)	H25B—C25—H25C	109.5
O1—C2—C1	116.3 (16)	N3—C26—C27	124.9 (16)
C3—C2—C1	121.2 (15)	N3—C26—H26	117.6
C2—C3—C4	124.5 (15)	C27—C26—H26	117.6
C2—C3—H3	117.8	C26—C27—C28	120.8 (18)
C4—C3—H3	117.8	C26—C27—Cl4	119.4 (14)
O2—C4—C3	122.4 (17)	C28—C27—Cl4	119.4 (14)
O2—C4—C5	118.3 (17)	N4—C28—C27	122.7 (17)
C3—C4—C5	118.2 (17)	N4—C28—C29	122.2 (17)
C4—C5—H5A	109.5	C27—C28—C29	115.0 (16)
C4—C5—H5B	109.5	C30—C29—C28	120.6 (16)
H5A—C5—H5B	109.5	C30—C29—Cl3	118.5 (15)
C4—C5—H5C	109.5	C28—C29—Cl3	120.8 (13)
H5A—C5—H5C	109.5	N3—C30—C29	122.8 (17)
H5B—C5—H5C	109.5	N3—C30—H30	118.6
C7—C6—H6A	109.5	C29—C30—H30	118.6
C7—C6—H6B	109.5	O13—C31—H31A	109.5
H6A—C6—H6B	109.5	O13—C31—H31B	109.5
C7—C6—H6C	109.5	H31A—C31—H31B	109.5
H6A—C6—H6C	109.5	O13—C31—H31C	109.5
H6B—C6—H6C	109.5	H31A—C31—H31C	109.5
O3—C7—C8	121.9 (15)	H31B—C31—H31C	109.5
O3—C7—C6	111.4 (16)		

### (4-Amino-3,5-dichloropyridine-κN1)dioxidobis(pentane-2,4-dionato-κ2O,O')uranium(VI) methanol hemisolvate Hydrogen-bond geometry (Å, º)

**Table:**

*D*—H···*A*	*D*—H	H···*A*	*D*···*A*	*D*—H···*A*
N4—H4*B*···O13	0.86	2.09	2.90 (2)	157
